# Supplementary material for: Human cytomegalovirus-encoded US9 targets MAVS and STING signaling to evade type I interferon immune responses
Source: Nat Commun. 2018 Jan 9;9:125. doi: 10.1038/s41467-017-02624-8 (PMC5760629; doi:10.1038/s41467-017-02624-8)
Supplement: Supplementary file 1 — Supplementary Information [file 41467_2017_2624_MOESM1_ESM.pdf]

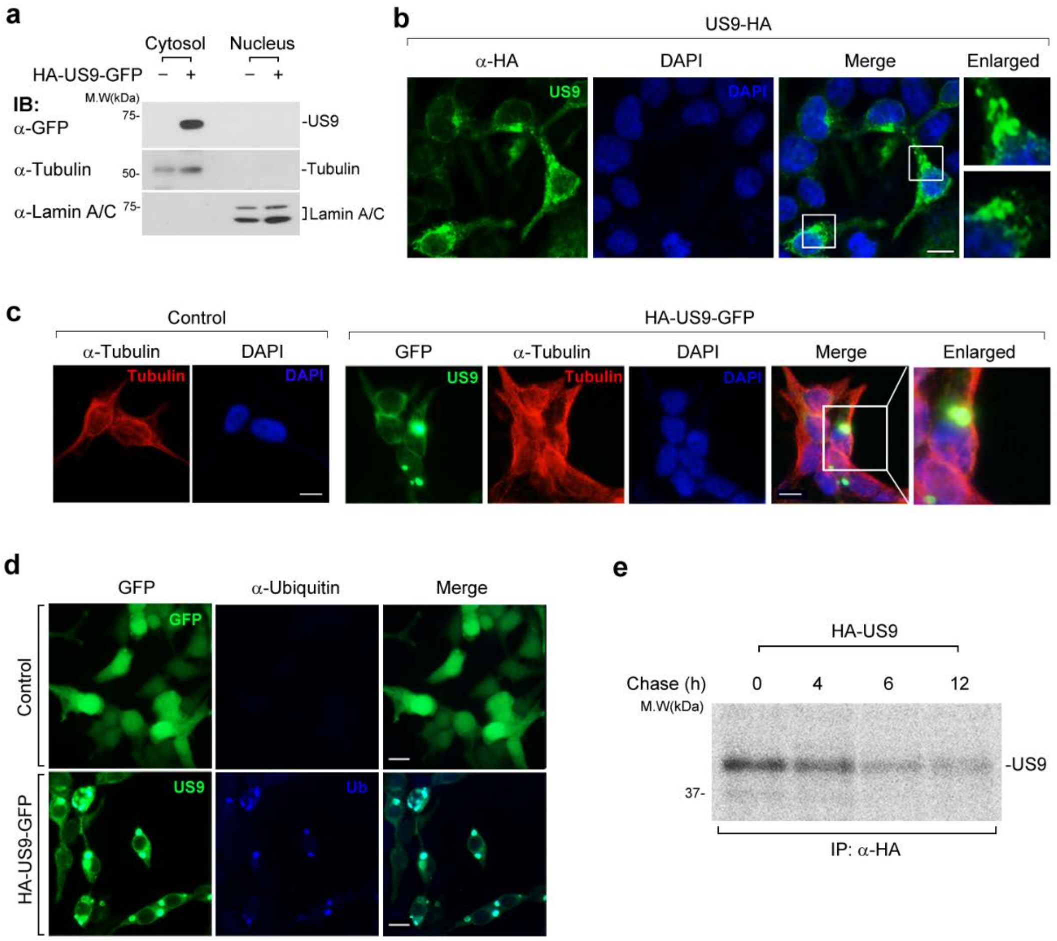

Supplementary Figure 1. US9 enriched foci with long half-life negatively relates MAVS-mediated IFN-β induction (a) US9 is not localized in the nucleus. Cytosolic and nuclear fractions isolated from HA-US9-GFP-expressing HEK293T cells were subjected to immunoblot analysis using the indicated antibodies. Anti-Tubulin and anti-Lamin A/C were used as the cytosolic and nuclear markers, respectively. **(b)** HEK293T cells expressing C-terminal HA-tagged US9 were stained with anti-HA antibody, followed by AlexaFluor 488-conjugated antibody. The nuclei were stained with DAPI. Scale bars, 10 µm. **(c)** HEK293T cells in the absence or presence of HA-US9-GFP were stained with cytoplasm marker (anti-Tubulin). The nuclei were stained with DAPI. Scale bars, 10 µm. **(d)** US9 colocalizes with ubiquitin. Control vector (pEGFP-N3)- or HA-US9-GFP-expressing HEK293T cells were immunostained with anti-Ub antibody, followed by AlexaFluor 350-conjugated antibody. Scale bars, 10 µm. **(e)** US9 has a long half-life. HA-US9-expressing HEK293T cells were labeled with [^{35}S] methionine/cysteine for 1 h and chased for indicated hours. Cells were lysed in 0.5% NP-40 and the supernatants were immunoprecipitated with anti-HA antibody. Data are representative of three independent experiments.

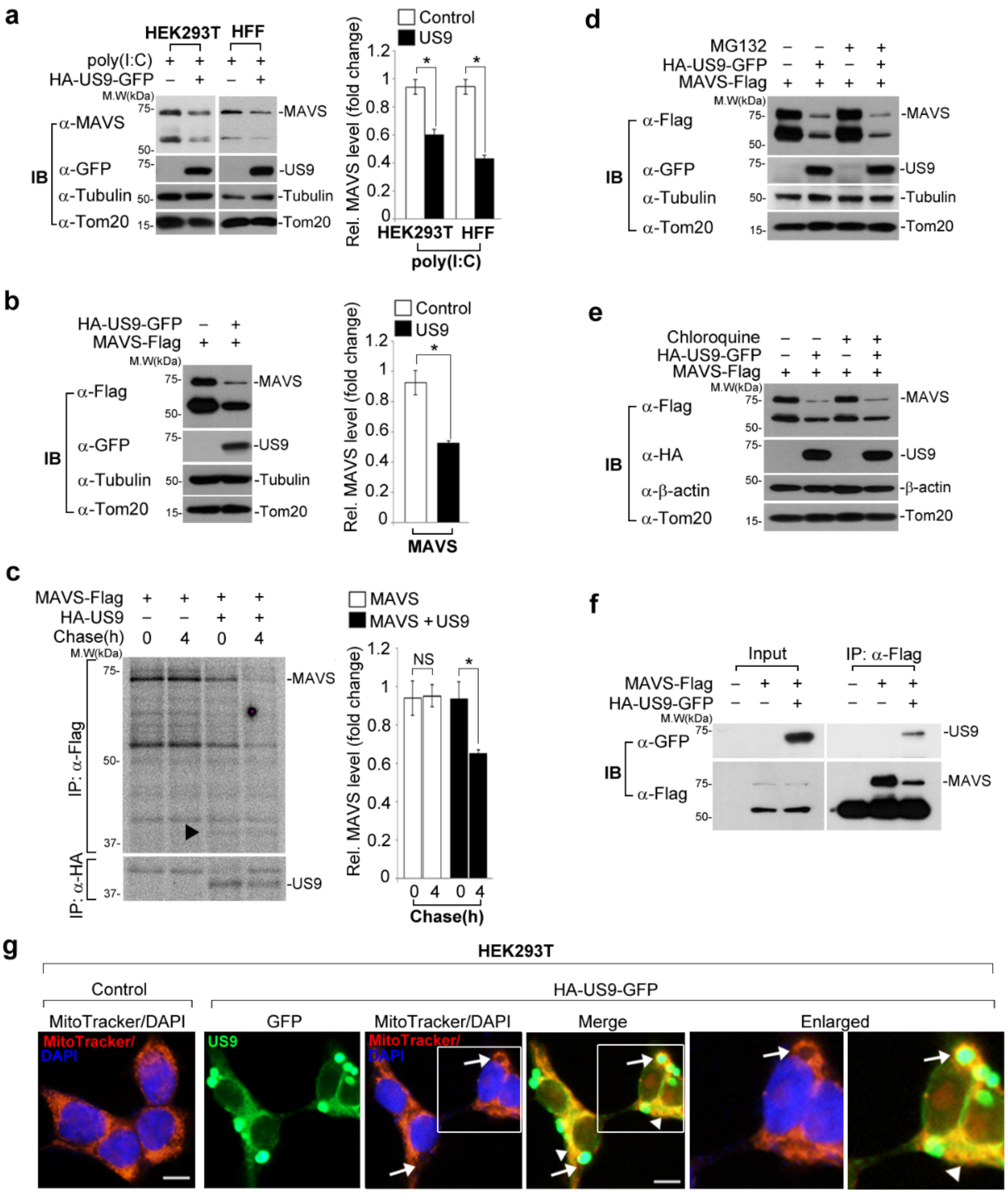

Supplementary Figure 2. US9 reduces MAVS levels in a proteasome- and lysosome-independent manner (a) US9-mediated endogenous MAVS downregulation in different cell types. HA-US9-GFP-expressing HEK293T and HFF cells transfected with poly(I:C) (10 μg/ml). Cell lysates were immunoblotted with the indicated antibodies. Right graph, quantification of MAVS levels relative to Tubulin. *$P<0.005$ (Student's *t*-test). **(b)** US9 downregulates MAVS levels. HEK293T cells expressing MAVS-Flag in the presence or absence of HA-US9-GFP were lysed in RIPA buffer and cell lysates were analyzed by immunoblot analysis with the indicated antibodies. Right graph, quantification of MAVS

levels relative to Tubulin. *P<0.005 (Student's t-test). **(c)** US9 destabilizes the half-life of MAVS. HEK293T cells that express MAVS-Flag and HA-US9 were labeled with [^{35}S] methionine/cysteine for 1 h and chased for 4 h. Cells were lysed, and then the supernatants were immunoprecipitated with anti-Flag antibody, followed by re-IP with anti-HA antibody. Right graph, quantification of the MAVS levels. *P<0.005 (Student's t-test). **(d and e)** US9-mediated MAVS reduction is independent of proteasome and lysosome pathways. HEK293T cells expressing MAVS-Flag and HA-US9-GFP were treated with MG132 (20 µM) **(d)** or chloroquine (100 µM) **(e)** for 4 h. Cells were lysed with RIPA buffer and then subjected to immunoblot analysis with the indicated antibodies. **(f)** US9 interacts with MAVS. HEK293T cells transfected with the indicated plasmids were lysed. Cell lysates were subjected to immunoprecipitation with anti-Flag antibody prior to immunoblot analysis with the indicated antibodies. **(g)** Mitochondrial US9 induces $\Delta\psi_m$ dissipation. HEK293T cells were transfected with mock vector or HA-US9-GFP and stained with MitoTracker Orange CMTMRos. Boxed images highlight regions with loss of $\Delta\psi_m$. The nuclei were stained with DAPI. Scale bars, 10 µm. Data are representative of three independent experiments and are presented as mean ± s.d. in **a**, **b** and **c**.

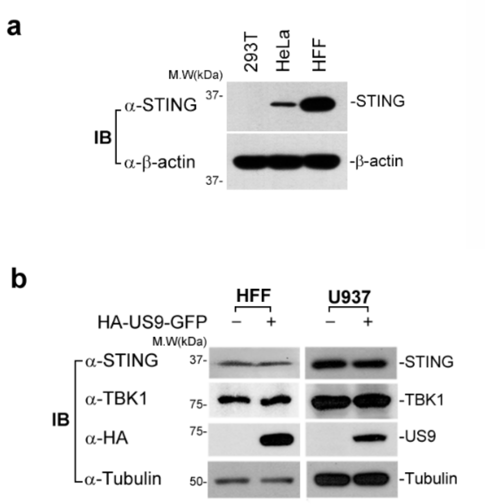

Supplementary Figure 3. US9 does not affect endogenous STING or TBK1 protein levels (a) Endogenous STING is strongly expressed in HFF cells. HEK293T, HeLa, and HFF cells were lysed and immunoblotted with the indicated antibodies. **(b)** HFF and U937 cells expressing control vector or HA-US9-GFP were lysed and analyzed by immunoblotting with the indicated antibodies. Data is representative of three independent experiments.

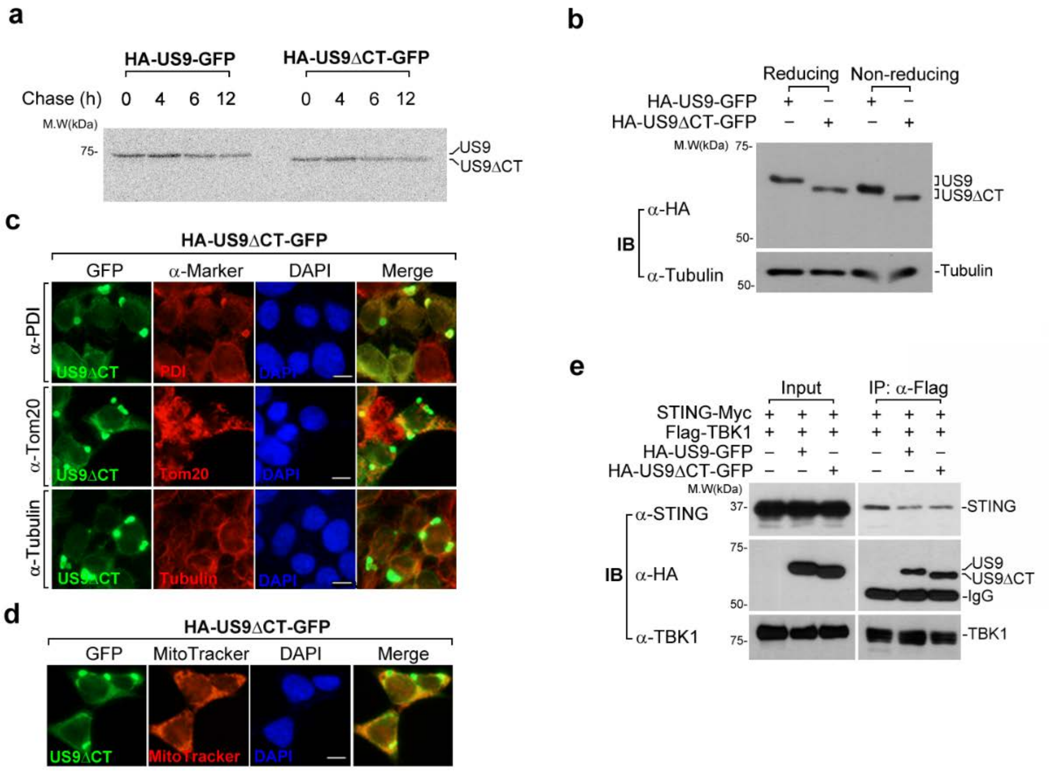

Supplementary Figure 4. Effects of US9 cytoplasmic domain on STING-TBK1 interaction (a) US9 and CT deletion mutant have similar half-lives. HEK293T cells expressing HA-US9-GFP or HA-US9ΔCT-GFP were labeled with [^{35}S] methionine/cysteine for 1 h and chased for 4 h. Cells were lysed, and then the supernatants were immunoprecipitated with anti-HA antibody. **(b)** HEK293T cells expressing HA-US9-GFP or HA-US9ΔCT-GFP were detected by immunoblot analysis under reducing and non-reducing conditions. **(c)** Subcellular localization of US9ΔCT. HEK293T cells were transfected with HA-US9ΔCT-GFP and immunostained with the indicated antibodies, followed by AlexaFluor 568-conjugated antibody. The nuclei were stained with DAPI. Scale bars, 10 µm. **(d)** The CT domain of US9 is dispensable for dissipating $\Delta\psi_m$. US9ΔCT expressing HEK293T cells were stained with MitoTracker Orange CMTMRos. The nuclei were stained with DAPI. Scale bars, 10 µm. **(e)** US9ΔCT slightly disrupts STING-TBK1 interaction. HEK293T cells expressing STING-Myc and Flag-TBK1 together with US9 or US9ΔCT were lysed. The lysates were immunoprecipitated with anti-Flag antibody prior to immunoblot analysis with the indicated antibodies. Data are representative of three independent experiments.

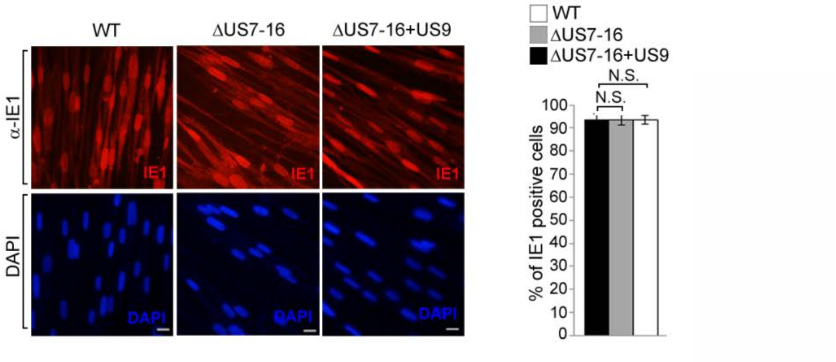

Supplementary Figure 5. Wild-type HCMV and mutant viruses, HCMVΔUS7-16 and HCMVΔUS7-16+US9, have similar infection efficiency HFF cells were seeded in 96-well plate and after 1day, cells were infected with virus strains (MOI 2). For the detection on IE1-positive cells, cells were stained with IE1 antibody and counted. Data are representative of three independent experiments and are presented as mean ± s.d.

Supplementary Figure 6. List of uncropped images of western blot analysis

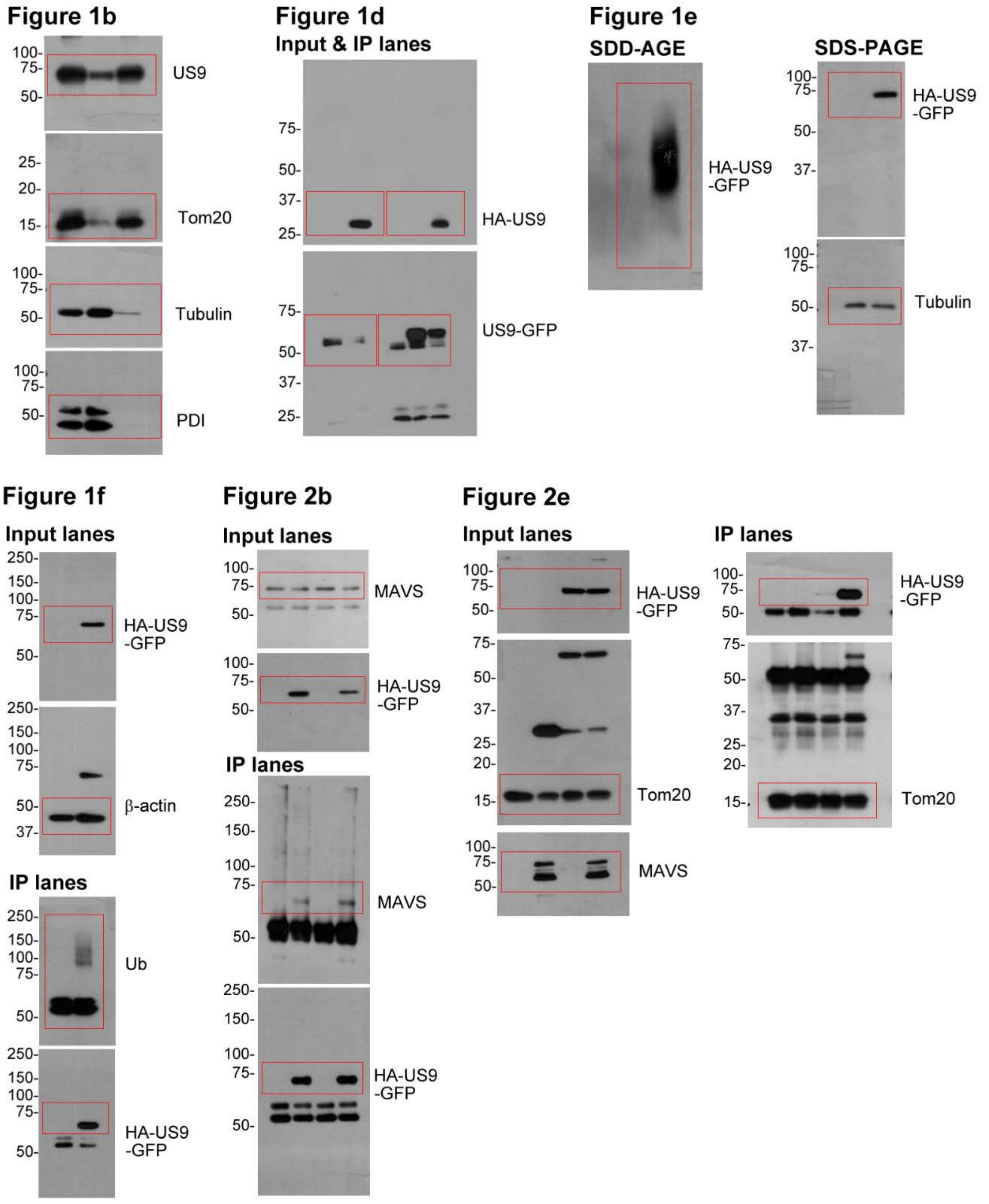

Figure 2f

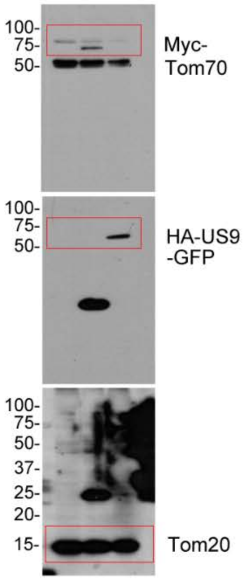

Figure 2g

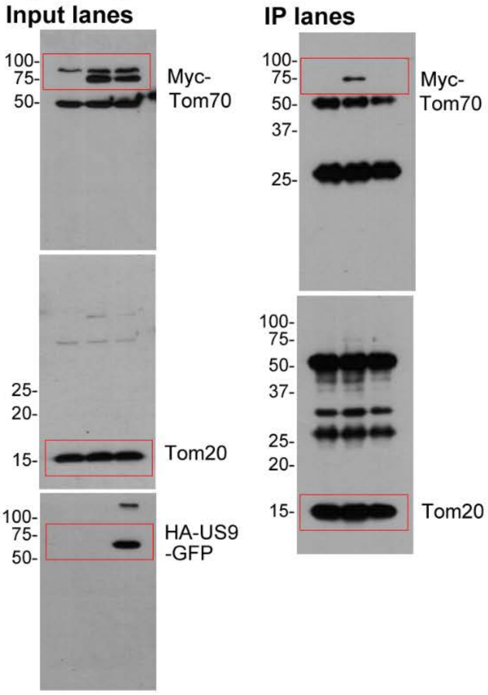

Figure 2i

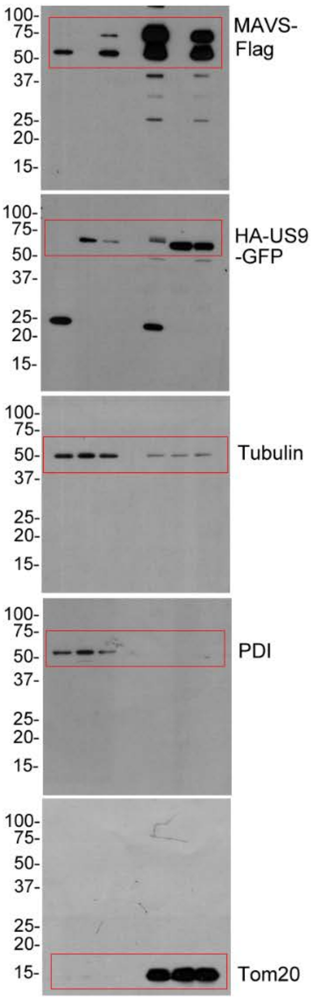

Figure 3d

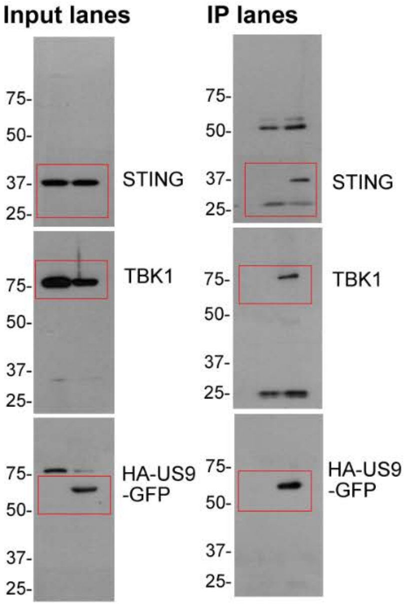

Figure 3e

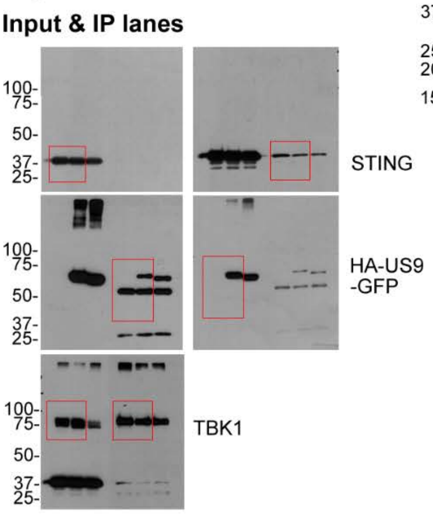

Figure 3f

SDS-PAGE **SDD-AGE**

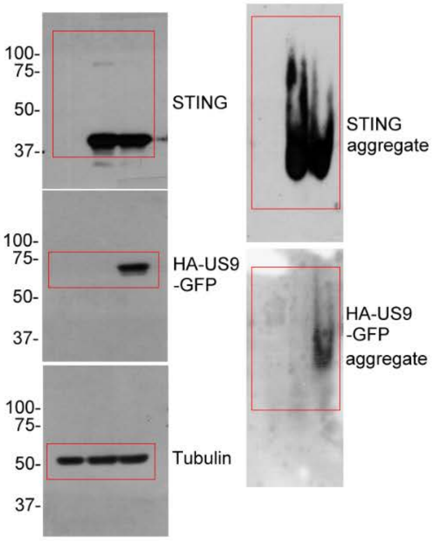

Figure 4c

Input lanes **IP lanes**

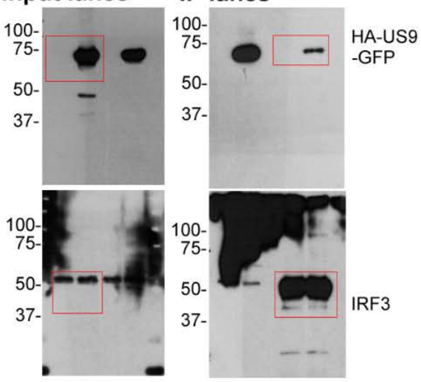

Figure 4d

MAVS-induced p-IRF3

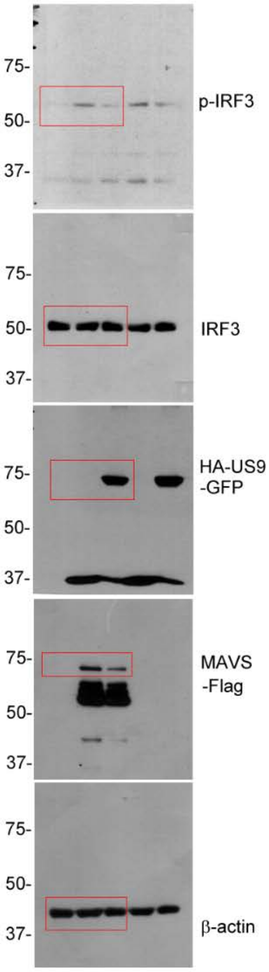

Figure 5c

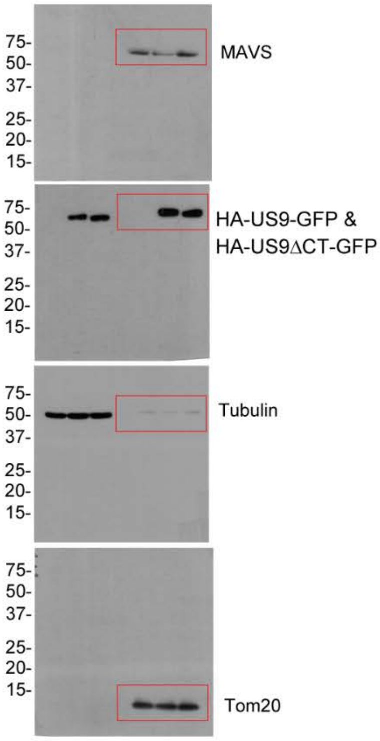

Figure 4d

STING-induced p-IRF3

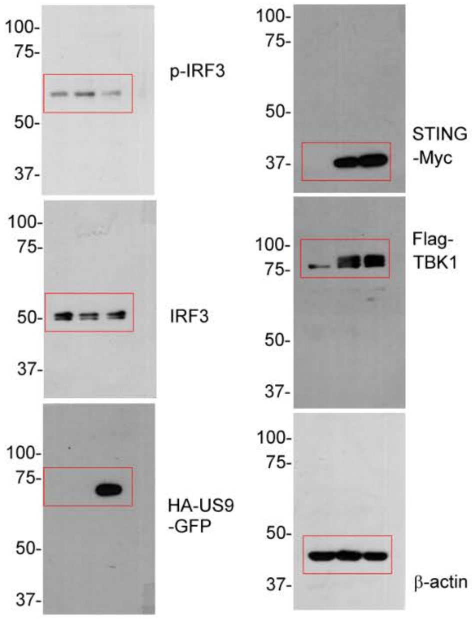

Figure 5e

Input & IP lanes

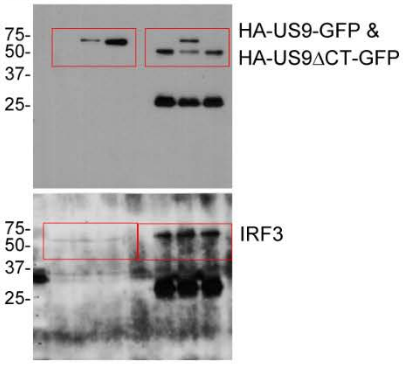

Figure 5g

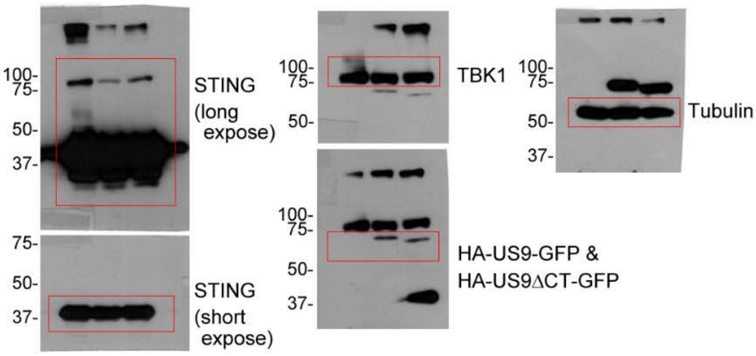

Figure 6g

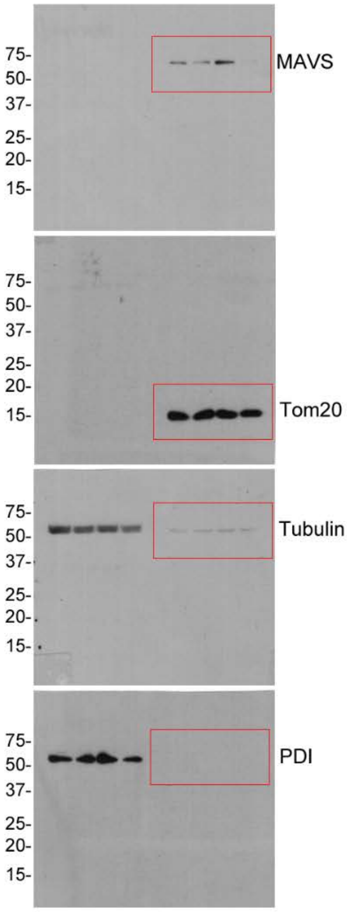

Figure 6h

Input & IP lanes

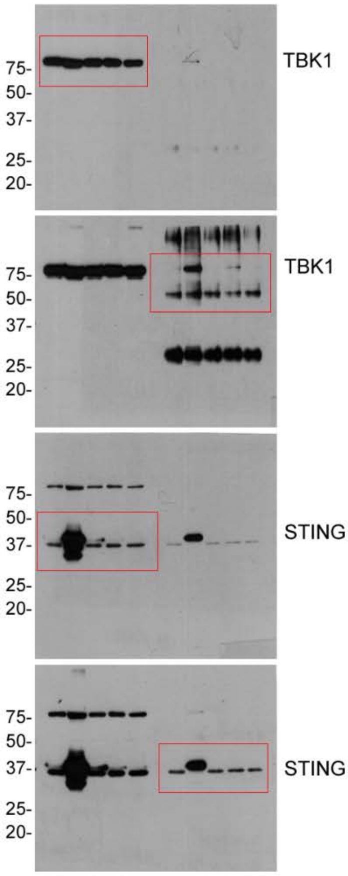

Figure 6i

Nuclear fraction

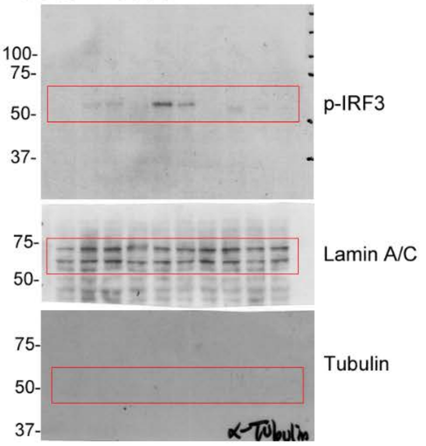

Cytosolic fraction

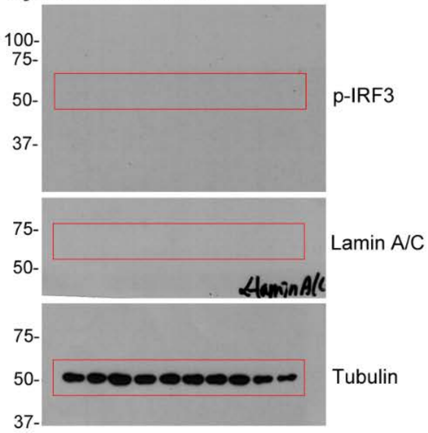

Supplementary Table, related to Figures 1, 5, and 6. Sequences of primers for RT-PCR

Gene mRNA	Forward Primer<br>Reverse Primer
#1: US9-1	5'- CGA CTC TCT TAC GTG ATG TTA -3'<br>5'- GAC ACC GAA GCT GAA CAA G -3'
#2: US9-2	5'- ATT CTC GAG AAG GAG TCG CTC CGG TTG TC -3'<br>5'- ATC TCT AGA TCA ATC GTC TTT AGC CTC TTC TTC C -3'
#3: US9ΔCT	5'- CGA CTC TCT TAC GTG ATG TTA -3'<br>5'- ATT AAG CTT GGT GCC GAC CTC GGA CCC A -3'
#4: IFN-β	5'- TGG AAT GAG ACT ATT GTT GAG AA -3'<br>5'- ATT TCC ACT CTG ACT ATG GTC -3'
#5: MAVS	5'- ATT CTC GAG CCG TTT GCT GAA GAC AAG ACC TAT AAG -3'<br>5'- GCA GGA AGT GAC GGT GGC TCC AGT GGG TCT GGG -3'
#6: STING	5'- ATT AAG CTT GCC ACC ATG CCC CAC TCC AGC CTG C -3'<br>5'- ACA GTC CAA TGG GAG GAG AA -3'
#7: TBK1	5'- ATT GGA TCC ATG CAG AGC -3'<br>5'- ATT ATC GAT CTA AAG ACA GTC AAC GTT -3'
#8: IE1	5'- CTG ATA ATC CTG ACG AGG GC -3'<br>5'- TGC TCC TTG ATT CTA TGC CG -3'
#9: GAPDH	5'- TGA TGA CAT CAA GAA GGT GGT GAA -3'<br>5'- TCC TTG GAG GCC ATG TGG GCC AT -3'
